# Up- regulation of miR-328-3p sensitizes non-small cell lung cancer to radiotherapy

**DOI:** 10.1038/srep31651

**Published:** 2016-08-17

**Authors:** Wei Ma, Chao-nan Ma, Nan-nan Zhou, Xian-dong Li, Yi-jie Zhang

**Affiliations:** 1 Department of Respiratory, Huaihe Hospital of Henan University, 475000, Henan, China

## Abstract

MicroRNAs (miRNAs) are believed to be resistant against radiotherapy in certain types of cancers. The aim of our study was to determine the clinical application of miRNAs in non-small cell lung cancer (NSCLC). Sixty NSCLC tissue samples and adjacent histologically normal tissues were obtained for miRNAs microarray analysis and validated by RT-qPCR. Correlation between miRNA expression level and clinicopathological features was evaluated. Our study examined the influence of changed miRNA expression on the damaged DNA and its associated radio sensitivity. Luciferase assay was performed to determine potential effects on the targeted gene. Our study identified fifteen altered miRNAs in which miR-328-3p was down regulated in NSCLC tumour tissue as compared to normal tissues. Down-expression of miR-328-3p was positively associated with an enhanced lymph node metastasis, advanced clinical stage and a shortened survival rate. miR-328-3p expression was decreased in A549 cells compared to other NSCLC cell lines. Up-regulation of miR-328-3p demonstrated a survival inhibition effect in A549 and restored NSCLC cells’ sensitivity to radio therapy. An increased miR-328-3p expression promoted irradiation-induced DNA damage in cells. γ-H2AX was identified as the direct target of miR-328-3p. Over-expressed miR-328-3p can improve the radiosensitvity of cells by altering the DNA damage/repair signalling pathways in NSCLC.

Non-small cell lung cancer (NSCLC) is a major histopathological type of lung cancer, and most fatalities among cancer patients are caused by NSCLC^1^. Several research findings have provided substantial benefit to the treatment for lung cancer patients. However, the five year survival rate poses a barrier towards effective prevention and treatment of this condition^2^. Radiotherapy is often the primary line of treatment for lung cancer, but some patients have demonstrated resistance to radiotherapy despite possessing similar age, gender and life factors^3^. Clinicians believe in restoring cell radiation sensitivity because of its potential benefit in treating this condition^4^,^5^. Despite the advancements in cellular radiosensitive biology such as cell apoptosis, cell cycle and DNA repair, early molecular therapeutic markers for radio resistance still requires thorough research for the management of lung cancer^6^,^7^,^8^. It is commonly accepted that restoring cell radiation sensitivity can provide a favourable outcome for lung cancer patients.

Endogenous microRNAs (miRNAs) are a group of short non-coding RNA molecules that regulate gene transcription levels in radiation response processes. Since irradiation alters the DNA by inducing breaks in its structure, the involved repair mechanism pathways could affect cellular radiosensitivity^9^,^10^,^11^. As a result of irradiation, the repair sensors in DNA such as ATM and histone H2AX phosphorylate are activated and form DNA repair effector protein complexes by recruiting DNA- dependent protein kinases. Consequently, blocking the repair process alters the mitotic phase and leads to cell death, which can lead to radiosensitvity in cancer cells^12^,^13^. Previous studies have shown that miR-421 and miR-24 prevents DNA repair response by downregulating ATM and H2AX expression hence leads to an increased IR-induced genomic instability and apoptosis *in vitro*^14^,^15^. As a result, it can be hypothesized that over/under expression of miRNA may cause cancer cells to become resistant or sensitive to radiation therapy, which depends on DNA damage. Therefore, more research needs to be undertaken to understand and assess the role of miRNA in DNA damage. Our study aims to determine the role of miRNAs and its response in the regulation of DNA damage in NSCLC. Our study found that down expression of miR-328-3p was an unfavourable predictor of clinical outcome in NSCLC patients. Up-regulation of miR-328-3p demonstrated a survival inhibition effect in A549 and restored NSCLC cell sensitivity to radio therapy via alteration in the expression of γ-H2AX.

## Results

### Association of differential miRNA expression and clinical features in NSCLC

**Figure 1:**
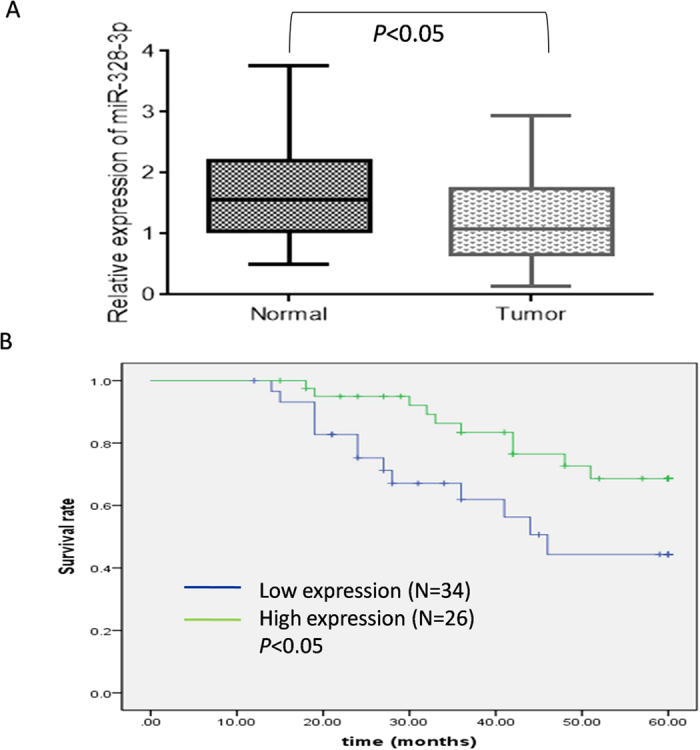
Comparison of miRNA expression profiles in NSCLC and paired normal tissues. (**A**) miR-328-3p expression level was significantly lowered in NSCLC tissues compared to the adjacent non-cancerous tissues. U6 was used as an internal control. *p < 0.05. (**B**) Shortly, overall survival time was observed in patients with a low miR-328-3p expression compared to a higher miR-328-3p expression patients as shown by the Kaplan-Meier survival curve (*p* < 0.05).

A total of 15 miRNAs (8 up-regulated and 7 down-regulated) were differentially expressed (changed in expression by >1.5-fold) in primary NSCLC cells and were compared with paired adjacent normal tissues (Supplementary Table S1). Among them, miR-328-3p exhibited the highest down regulation fold change (−2.62) in both microarray and in qRT-PCR process in NSCLC tissues as compared to the corresponding non-cancerous tissues (Fig. 1A, *p* < 0.05). Furthermore, using the median miR-328-3p expression in all patients as a cut off, the patients were divided into a high miR-328-3p expression group and a low miR-328-3p expression group. Down-expression of miR-328-3p was associated with an increased lymph node metastasis, advanced clinical stage of NSCLC and tumor differentiation (Table 1, *p* < 0.05). No significant difference was observed between miR-328-3p expression, age and sex. Kaplan-Meier survival curve showed a significantly shortened survival rate in patients with a lowered expression of miR-328-3p than those patients who had a high miR-328-3p expression (Fig. 1B, *p* = 0.024).

### Up- regulation of miRNA-328 indicates increased radiosensitivity in NSCLC cells

**Figure 2:**
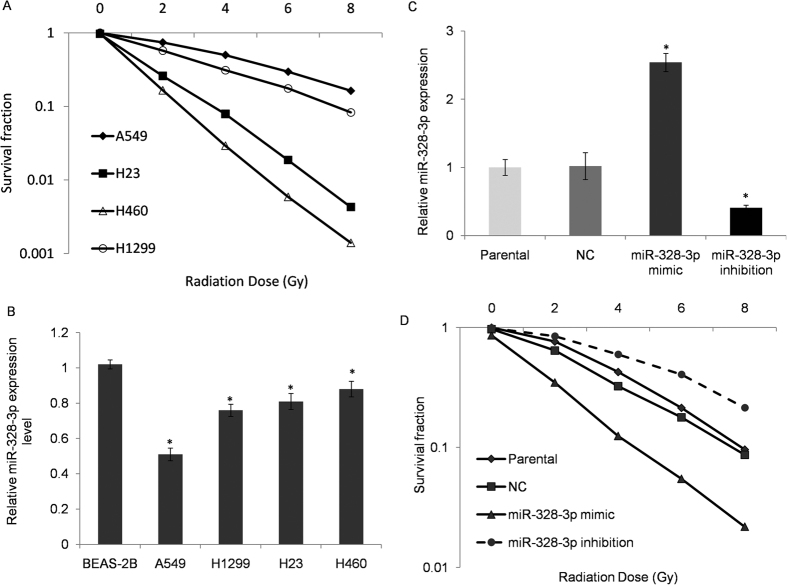
Comparison of the inner radiosensitivity and miR-328-3p expression in NSCLC cell lines. (**A**) Four NSCLC cell lines A549, H1299, H23 H460 and non-cancerous bronchial epithelial cells BEAS-2B were irradiated with a 0, 2, 4, 6, or 8 Gy and were performed with colony formation assay. Innate radio-sensitivity was observed as followed: A549>H23>H1299>H460. (**B**) miRNA-328 expression in four NSCLC cell lines was detected using the qRT-PCR. U6 served as an internal control. Relatively decreased expression of miR-328-3p was globally observed in all cell lines compared to normal epithelial cells. *p < 0.05. Results presented as the mean ± SEM of the values obtained in 3 independent experiments. (**C**) Transfection efficiency of miR-328-3p mimic and inhibitor verified with the RT-PCR. *p < 0.05 as compared to the negative control. (**D**) Irradiation response of A549 cell following transfection with a miR-328-3p mimic, inhibitor or control. miR-328-3p mimic sensitive cell response to irradiation; miR-328-3p inhibitor attenuated this effect.

To determine the role of miR-328-3p expression in radiosensitivity of NSCLC, we compared the inner radiosensitvity of four NSCLC cell lines through colony formation assay after exposing them to varying degrees of radiation (0, 2, 4, 6 and 8 Gy). Our data showed that H460 cells had the highest radiosensitvity while A549 was more resistant to irradiation than other cell lines (Fig. 2A, *p* < 0.05). Afterwards, we evaluated the expression of miR-328-3p in every cell line and observed a decreased expression of miRNA-328-3p in response to irradiation. In comparison with radiosensitive H460 cells, miR-328-3p in A549 cell line showed a decrease by 2.5 folds (Fig. 2B, *p* < 0.05). A549 cells were transfected with miR-328-3p mimic, inhibitor and a negative control to further confirm whether miR-328-3p actually enhanced the sensitivity of A549 cell lines to irradiation. Transfection efficiency was validated using the RT-PCR (Fig. 2C, *p* < 0.05). After 48 hours of transfection, the cells were exposed to various doses of IR (0, 2, 4, 6, and 8 Gy), and we performed a survival fraction assay. Our results showed that up- regulation of miR-328-3p significantly inhibited the survival fraction as compared to the negative control group. The up-regulation had a close relation with the doses of radiation while inhibition of miR-328-3p attenuated this effect (Fig. 2D).

### Up regulation of miR-328-3p increased radio sensitivity and promoted DNA damage in A549 cells

**Figure 3:**
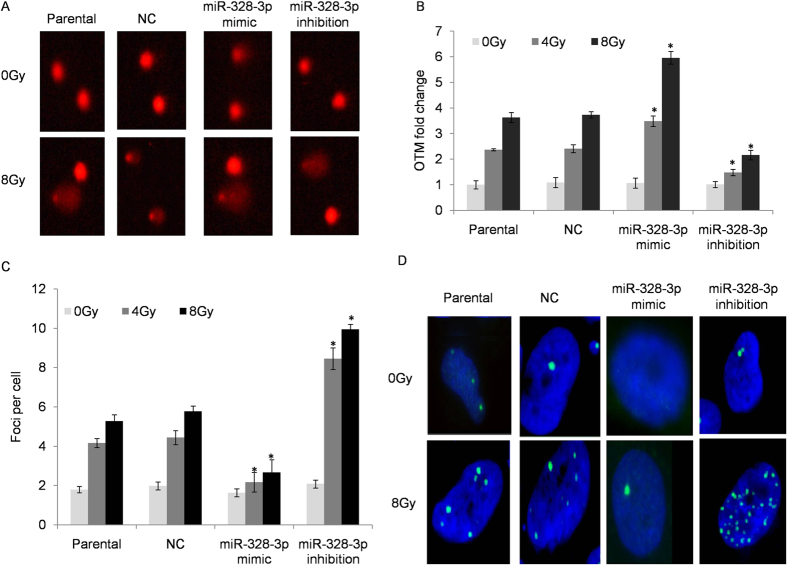
miR-328-3p over-expression increased radio sensitivity and promoted DNA damage of A549 cells. (**A,B**) Comet assay was performed to evaluate DNA damage response following miR-328-3p transfection. ANOVA test was used to determine the statistical significance of differences among the groups. Results presented as mean ± SEM of values obtained in three independent experiments. (**C,D**) Blue represents stained nuclei and green represents stained γ-H2AX foci. Mean number of foci per cell for various doses shown after 0 to 8 Gy of irradiation. *p < 0.05, compared to the parental control.

The key mechanism of radiotherapy is to induce DNA double strand breaks. To further explore the influence of miRNA-328-3p on DNA damage response to ionizing radiation, we investigated the indicators inducing the double-strand break (DSB) following a different miRNA-328-3p treatment. Comet assay results indicated that over-expression of miR-328-3p could make cells more likely to suffer from DNA double break damage following irradiation (increased by 1.51 fold at 4 Gy and 1.84 fold at 8 Gy) as compared to the control transfected cells while inhibition of miR-328-3p partially reversed this effect (Fig. 3A,B). Similarly, the foci formation of γ-H2AX also demonstrated a significant repression effect of miR-328-3p (decreased by 1.91 fold at 4 Gy and 1.98 fold at 8 Gy) as compared to negative controls (Fig. 3C,D).

### Identification of the direct target of miR-328-3p and its influence on target genes

**Figure 4:**
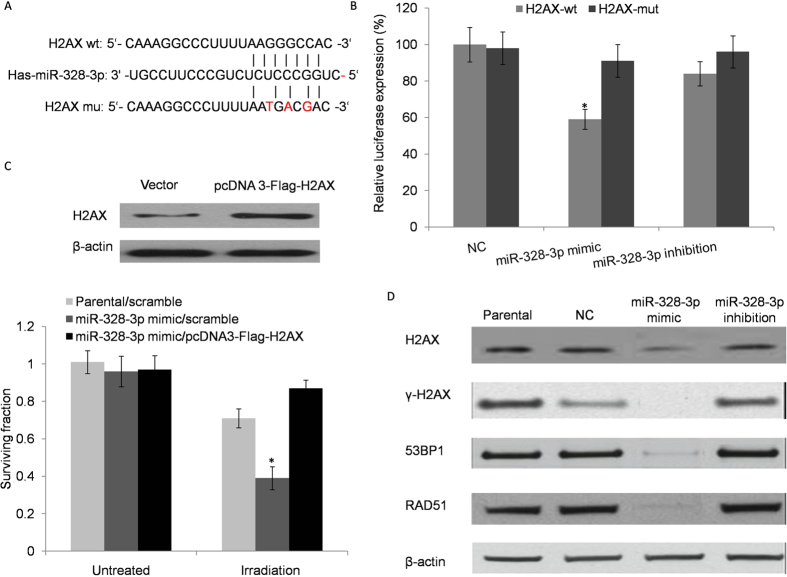
Identification of miR-328-3p directly to the target gene and its influence. (**A**) miR-328-3p binding to its site in the target gene H2AX. (**B**) Comparison of luciferase activity in vectors containing H2AX wild or mutation binding sequences. (**C**) Transient over expression of H2AX reversed the radiation sensitizing effect of miR-328-3p mimic in A549 cells with irradiation treatments. *p < 0.05. (**D**) Western blot analysis of DSB marker proteins γ-H2AX, H2AX, 53BP1 and RAD51 in A549 cells with miR-328-3p in transfected and non-transfected cells before and after exposure to 8-Gy X-ray irradiation. β-actin used as an internal control. Each experiment was performed in triplicate. Relative expression level demonstrated as gel images below.

To understand the molecular mechanism of miR-328-3p and how it affects malignant development of NSCLC, we searched for potential mRNA targets of miR-328-3p using three prediction online tools (miRanda, Diana mirPathway, and TargetScan). We identified a putative miR-328-3p-binding site in the 3′-UTR of H2AX mRNA in all of the three databases. Other predicted potential target genes, which are involved in tumour or cell function, are listed (Supplementary Table S2). A luciferase reporter assay, which contained wild and mutant binding sequence of 3′-UTR of H2AX mRNA, was generated in our study (Fig. 4A). Luciferase activity indicated that miR-328-3p inhibited H2AX, which contained a wild binding sequence signal that was compared with the miR-NC negative control (Fig. 4B), but had no effect on the activity of mutation reporter vector. The luciferase assay suggested that miR-328-3p interacts directly with the 3′-UTR of H2AX’s mRNA. To determine the importance of H2AX in the regulatory role of miR-328-3p in radiation response, we transiently over expressed H2AX in cells transfected with miR-328-3p mimic, and the expression of γ-H2AX was evaluated by the RT-PCR. At 48 hours after transfection, the cells were subjected to 8-Gy of radiation and were collected 48 hours after irradiation. As shown in Fig. 4C, an up regulation of miR-328-3p sensitized the cell response to radiation (increased by 2.89 fold at 8 Gy) as compared to the control, and an over expression of H2AX eliminated this sensitizing effect. Rapid phosphorylation of histone H2AX to γ-H2AX is a biomarker of DNA double-strand breaks induced by ionizing radiation. This phosphorylation accumulates numerous signalling and repair proteins, such as ATM, γ-H2AX, 53BP1 and Rad51 at DNA breaks to form discrete foci. To investigate the influence of miR-328-3p on these DSB marker proteins, we measured their expression levels in A549 cells transfected with miR-328-3p mimic or inhibitor after the DSB proteins were exposed to 8-Gy X-ray irradiation. Our results revealed that miR-328-3p decreased the protein expression level of H2AX, γ-H2AX, 53BP1 and Rad51 following the radiation treatment as compared to the normal control (Fig. 4D). Taken together, these results demonstrated that miR-328-3p enhanced irradiation-induced DNA double-strand breaks in lung cancer cells by regulating the expression of DSB sensor proteins.

### Up-expression of miRNA-328-3p increases radiation sensitivity *in vivo*

**Figure 5:**
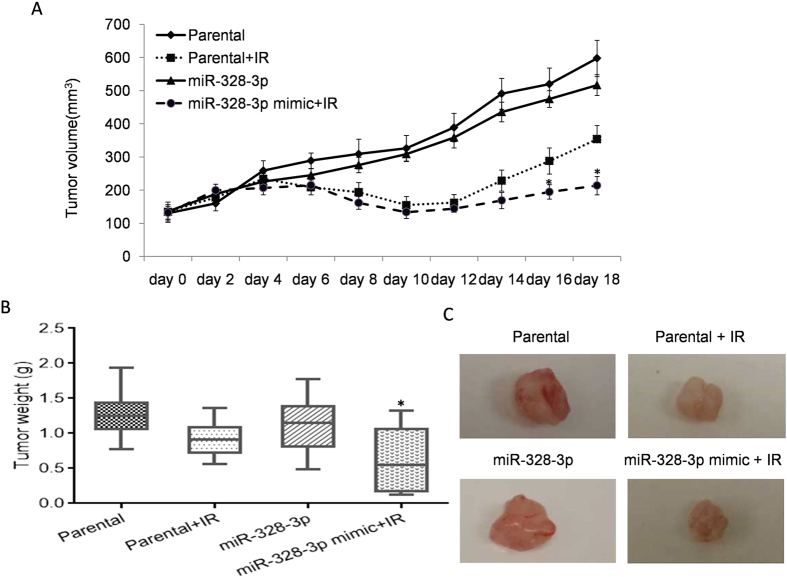
miR-328-3p sensitive tumour radiation response *in vivo*. (**A**) Nude rats (n = 10 each group) were subcutaneously injected with cells carrying miR-328-3p mimic or control. Tumour volumes were measured every two days. Following 8 Gy irradiation, over-expression of miR-328-3p significantly inhibited tumour growth compared to the parental cells. *p < 0.05 compared to the parental control with an IR. (**B**) Quantitative summary of the tumour weights. Data presented as mean ± SEM of the three independent experiments. *p < 0.05 compared to the parental control. (**C**) Characteristic image of tumours obtained from the xenograft model after exposure to irradiation.

Based on the previous clinical data and experimental results derived *in vitro*, we investigated the influence of miR-328-3p on radio-sensitivity in a xenograft model. To setup a xenograft model, 1 × 10^5^ cells (control or miR-328-3p mimic cells) were injected subcutaneously into the right flanks of rats (n = 10 each group). Rat models accepted 8 Gy radiation when the average tumour volume reached 200 mm^3^ at day 6. After 12 days of irradiation exposure, tumours were collected, and the tumour weights were measured. Significantly decreased tumour volumes were observed after exposure to 8 Gy irradiation in both parental and miR-328-3p over expressed cells on day 6. From day 14, tumour volume started to slightly increase in both cells, and miR-328-3p over expressed cells exhibited a lowered tumour volume compared to the parental cells (Fig. 5A). Tumours were removed and measured at the end of the study. As shown in Fig. 5B,C, over expression of miR-328-3p significantly repressed tumour growth.

## Discussion

Over the years, miRNA has gathered attention among researchers as a potential therapeutic option because of its ability to target multiple oncogenic pathways^16^,^17^. The regulatory role of miRNA in lung cancer and its potential diagnostic and prognostic implications has been greatly studied. Some of these studies have also reported the influence of miRNA on radiosensitivity management in cancer therapies^18^,^19^,^20^. Our data indicated a signature of fifteen altered miRNAs expression in response to clinical radiotherapy for NSCLC patients. 8 up-regulations (miR-95, miR-66, miR-335, miR-181-3p, miR-324, miR-126, miR-24, miR-787) and 7 down-regulations (miR-328-3p, miR-155, miR-7, miR-483-3p, let-7g, miR-505, miR-200c) revealed multiple roles of miRNAs in lung cancer tumorigenesis and progression. In our study, a significant number of down regulated miRNA observed were previously reported as tumor suppressors^21^,^22^,^23^ and commonly inactivated in non-small cell lung cancer^24^,^25^. Some up regulated genes, as demonstrated for miR-95 and miR-24, were found to be associated with a risk for lung cancer and a poor clinical outcome^26^,^27^. These miRNAs were also found to target different mRNAs involved in processes aberrant in tumorigenesis such as proliferation, survival, and differentiation. However, some significantly changed miRNAs have not been identified to participate in breast cancer, and little is known about the biological function and targeted genes of these miRNAs in NSCLC. Among them, miR-328-3p exhibited the lowest expression in our cohorts studied. Our analysis on miR-328-3p revealed that down regulation of this mRNA was closely linked with a decreased survival rate, an enhanced lymph-node invasion with an advanced clinical stage in tumour tissues. Study conducted by Wang *et al.* demonstrated thirty-nine cohorts who had non-small cell lung cancer, and these patients underwent a prospective RT. Patients were defined as radiosensitive and/or radio resistant based on the clinical outcome obtained such as the overall survival and the recurrence rate. Their research suggested that five upregulated miRNAs and seven downregulated miRNAs were present compared to the IR resistant group^28^. Previous study has provided evidence that miRNA-328 could decrease chemoresistance in glioblastoma cancer cells and breast cancer cells by down-regulating the ABCG2 gene^29^,^30^. Another study has observed the down-regulation of miR-328-3p in colorectal cancer patients. In addition, miR-328-3p over expression reversed the process of drug resistance and inhibited cell invasion of colon rectal cancer (CRC) cells^31^. Low expression of circulating microRNA-328 is reported to be associated with a poor prognosis in patients with acute myeloid leukemia (AML)^32^. Our results have added valuable evidence into the specimen database, which focused on miRNA and lung cancer. Due to limited human trials, more research has been published on *in vitro* studies instead of focusing on specific miRNAs and their pathways with radiosensitivity. For instance, a study confirmed a close association between the up-regulation of let-7 family and increased radiosensitvity through the K-Ras pathway^33^,^34^,^35^. Chen *et al.* reported that over expression of miR-101 was able to radiosensitize NSCLC cells, especially the cells with lower miR-101 levels, which highlights the use of miRNA as a therapeutic tool and requires adequate attention to the baseline endogenous level of miRNA^36^. Shin and his team demonstrated eight miRNAs (miR-345, miR-885-3p, miR-206, miR-516a-5p, miR-16-2, miR-106a, miR-48c-3p and miR-127-3p), which became altered in A549 cells in response to 20 and 40 Gyirradiation^37^.

Some studies have provided evidence on the regulatory role of specific miRNAs in DNA damage and repair pathways^38^,^39^,^40^. Over expression of miR-449a was observed to be associated with an increased radiosensitization due to DNA damage, apoptosis and altered cell cycle distribution in two lung cancer cell lines at a ranged dose (0–10 Gy)^41^. Similar cell biological alterations were also reported by Di Francesco for miR-27a in A549 cells after 2 Gy γ-irradiation exposure^42^. miR-421 was observed to induce an S phase checkpoint defect via suppression of the expression of ATM^39^. miR-16 controls Cdc25A and Wip1 phosphatise following DNA damage stimulation, thereby disturbs the ATM/ATR pathway^39^,^40^. We found that ectopic miR-328-3p expression could induce cell DNA damage after irradiation in A549 cell line, and miR-328-3p directly targeted the H2AX 3′-UTR and reduced the H2AX expression. Our study identified miR-328-3p as a negative regulator of γ-H2AX formation in response to irradiation treatment. Furthermore, the binding effect induced genomic instability after DNA damage and sensitized cells to radio therapy. γ-H2AX is recognized for its crucial role in the repair of DNA lesions by recruiting DNA damage signalling and repair proteins. Previous studies have found that H2AX-deficient cells are sensitive to IR and exhibit genomic instability, double-strand break repair defects and mild DNA damage checkpoint dysfunction^43^,^44^. It was observed that miR-138 acted as a DNA damage response (DDR) machinery factor to down regulate the IR induced histone H2AX phosphorylation and nuclear foci formation at the sites of DNA damage^45^. miR-24has been reported to directly downregulate H2AX expression in hematopoietic cells, to inhibit DNA damage repair response and to enhance chemosensitivity^34^. Taking all the previous research into perspective and the results of our study, it can be concluded that miRNAs controls DNA damage response and are responsible for modulating genomic stability and tumorigenesis.

To conclude, our study suggested that over expression of miR-328-3p sensitizes lung cancer cells to radiotherapy; miR-328-3p could potentially serve as a therapeutic target for lung cancer treatment. Despite the evidence available on the potential therapeutic value of miRNAs, further clinical studies on the use of miRNAs needs to be explored. Future studies could evaluate the role of miR-328-3p for treating other types of cancers.

## Material and Methods

### Clinical specimens

Sixty patients, who were diagnosed with histologically confirmed NSCLC, were recruited from our hospital between 2010 and 2015. A letter of consent was obtained from each cohort in this study. Lung tissues and adjacent non-tumor control tissues were obtained from the same surgery, and histological features of the specimens obtained were evaluated by two senior pathologists, according to the classification criteria from the World Health Organization (WHO)^46^. Samples were frozen in liquid nitrogen and then stored at −80 °C until use.

### Ethics statement

The study protocol was approved by the Ethics Committee of Henan University. All experimental procedures were carried out in accordance with the approved guidelines. Informed consent was obtained from all patients prior to surgery. Specimens were handled and carried out in accordance with the approved guidelines.

### Cell culture

Experimental lung cancer cell lines A549, H23, H460, H1299 and non-cancerous bronchial epithelial cells BEAS-2B were purchased from ATCC (ATCC, USA) and cultured in DMEM supplied with10% fetal bovine serum (Gibco, Carlsbad, CA, USA), 100 units/ml penicillin and 100 μg/ml streptomycin; the specimens were kept in humidified incubator at 37 °C with 5% CO_2_ until log phase in proliferation was obtained.

### Western blot analysis

Proteins samples were obtained with a lysis buffer containing protease/phosphatase inhibitor and separated with the SDS-PAGE gel. Semi-dry gel system was used to transfer protein samples to a nitrocellulose membrane. Following a PBS washing done twice, the membrane was incubated with primary antibodies including anti-γ-H2AX, H2AX, 53BP1 and RAD51 (Santa Cruz) at 4 °C overnight and then incubated with a second antibody (Santa Cruz). β-actin was chosen as an internal control. Protein images were observed with an ECL solution under an imaging analysis software.

### Real-time RT-PCR

Total RNAs were extracted using the Trizol reagent (Invitrogen). Ten nanograms of RNA was reverse transcribed using the Taqman miRNA Reverse Transcription Kit (Applied Biosystems) with a miRNA-specific primer. For H2AX gene expression, 100 ng of total RNA was reverse transcribed with a random hexamer. The Taqman miRNA Assay Kit or Gene Expression Kit (Applied Biosystems) was used for a quantitative PCR reaction on the ABI 7900 (Applied Biosystems) in a 96-well plate according to the manufacturer’s instructions. The differential expression was evaluated with the 2^−(∆∆Ct)^ method. RNU6B was chosen as an internal control.

### Cell transfection

For the transfection, 1 × 10^6 ^cells were treated with a 50 nM miR-328-3p mimic, inhibitor or a negative control (Shanghai GenePharma) in 1 μl of LipofectamineTM2000 (Invitrogen, USA) following the product guidelines. A commercial siRNA kit was used to repress H2AX protein expression (Invitrogen, USA). siRNA transfection was conducted with the Lipofectamine RNAi Max reagent (Invitrogen, USA) as previously described^47^.

### Over expression of H2AX

Human histone H2AX cDNA was obtained by the PCR from a human mammary gland cDNA library. The PCR product was cloned into a Flag-pcDNA3 (Invitrogen). Generation of stable H2AX-overexpressed cell lines, which followed the procedure described previously^48^. Cells were first transfected with pcDNA3-Flag-H2AX for 72 hours and then seeded at a low density. Stable transfectants were positively selected using neomycin at concentrations of 500 μg/ml. Stable transfectants were cultured in neomycin-containing media and were transferred to 96-well plates until they become visible. Stable transfectants were subsequently screened for H2AX overexpression through the Western Blot analysis.

### Dual-luciferase reporter assay

A putative 3′-UTR sequence of H2AX was site-directed mutant at two single bases using the QuickChange II site - directed mutagenesis kit (Stratagene, La Jolla, California, USA). Wild-type and mutant nucleotide sequences were inserted into the vector (Promega, Madison, WI, USA) to construct a luciferase reporter plasmid following the manufacturer’s instructions and using a previous report^49^. A549 cells were seeded in 96-well plates and transfected with a 50 nM miR-328-3p mimic, inhibitor or a negative control along with a 200 ng of established luciferase reporter plasmid using the LipofectamineTM2000 (Invitrogen). Cells were incubated for 48 hours and later collected to measure their luciferase activity using the dual-luciferase reporter assay (Promega).

### Survival fraction assay

Cell irradiation response was determined by a colony-forming assay. Briefly, 1 × 10^5^ cells were plated into a 6-well plate and transfected with a miR-328-3p mimic, inhibitor or a negative control. Plate was incubated for 10 days until the formation of colonies. The colonies were fixed, stained with crystal violet and counted using a microscope. A population of >50 cells was counted as one colony. Plating efficiency (PE) and surviving fraction (SF) were calculated following the equation: PE = number of colonies formed/number of cells seeded x 100%; SF = no. of colonies formed after treatment/no. of cells seeded x PE.

### Radiation treatment

Cells were seeded into 96-well plates and then treated with a range of radiation doses (0, 2, 4, 6, 8 Gy) using 6-MV X-rays (2100EX, Varian).

### Comet assay

1 × 10^5^ A549 cells (miR-328-3p mimics, negative control or miR-328-3p inhibitor) were seeded on a T25 culture flask to achieve a 70% confluence. Single-cell gel electrophoresis (Comet assay) was performed to assess the irradiation induced DNA double strand breaksmiR-328-3p^50^. Olive Tail Moment (OTM) was measured and scored in randomly selected 500 cells using a fluorescence microscope with the KOMET 5.0 software (Kinetic Imagine).

### γ-H2AX foci formation

γ-H2AX foci formation was investigated in cells following a differential treatment (miR-328-3p mimic, inhibitor or negative control) by using a fluorescence microscope (Nikon, Tokyo, Japan). Briefly, paraformaldehyde fixed cells were incubated with a mouse monoclonal primary antibody specific for Ser 139 phosphorylation of H2AX overnight at 4 °C and were continually incubated with the Alexa Fluor 488-labelled secondary antibody (Invitrogen, USA) for an additional 2 hours. Vectorshield® mounting medium for fluorescent microscopy (Vector Laboratories, Burlingame, CA, USA) and a cover slip were placed on top of the cells and later sealed with clear nail varnish.

### Mouse model

Forty male Sprague Dawley rats (n = 10 for each treatment group, average weight = 230 g) were obtained from the animal center of Henan University. Protocol for animal experimentation was approved by the Animal Research Committee of Henan University, and laboratory animal care as well as the user guide were followed. Rats were taken care of according to the laboratory animal care guidelines. To setup a xenograft model, 1 × 10^5 ^cells (control or miR-328-3p mimic cells) were injected subcutaneously into the right flanks of rats.

Tumour volume was measured every 2 days and calculated using the equation as previously described: (volume = length × width^2^/2)^20^. Rat models started to accept 8 Gy radiation exposure when the average tumour volume reached 200 mm^3^ at day 6. After 12 days of irradiation exposure, tumours were collected and their weights were measured.

### Statistical analysis

All data was presented as the mean ± standard error of mean (SEM) and analyzed by the SPSS 13.0 (SPSS Inc., Chicago, IL, USA). An ANOVA test was used to determine the statistical significance of differences among the groups. All data was derived from at least three independent experiments. A value of *P* < 0.05 was considered statistically significant.

## Additional Information

**How to cite this article**: Ma, W. *et al.* Up-regulation of miR-328-3p sensitizes non-small cell lung cancer to radiotherapy. *Sci. Rep.* **6**, 31651; doi: 10.1038/srep31651 (2016).

## Supplementary Material

Supplementary Information

## Figures and Tables

**Table 1:** Relationship between miR-328-3p expression and tumour clinicopathologic features.

Clinical and pathological features	N	miRNA-328-3p expression				
		Low	%	High	%	P value
All cases	60	34	56.7	26	43.3	
**Age**						0.205
<60	29	14	41.2	15	57.7	
>=60	31	20	58.8	11	42.3	
**Sex**						0.211
Female	42	26	76.5	16	61.5	
Male	18	8	23.5	10	38.5	
**Lymphatic metastasis**						0.030
Positive	22	18	52.9	4	15.4	
Negative	38	16	47.1	22	84.6	
**TNM stage**						<0.001
T1N0M0	21	7	20.6	14	53.8	
T2N0M0	24	13	38.2	11	42.4	
T3N0M0	15	14	41.2	1	3.8	
**Tumour differentiation**						0.018
Well	31	9	26.5	22	84.6	
Moderate	29	25	73.5	4	15.4	

Note: TNM, tumor node metastasis. T, size and/or extent of the primary tumor; N, spread to regional lymph nodes; M: the presence of metastasis. Data is presented as n (%).
