# Supplementary material for: Up- regulation of miR-328-3p sensitizes non-small cell lung cancer to radiotherapy
Source: Sci Rep. 2016 Aug 17;6:31651. doi: 10.1038/srep31651 (PMC4987701; doi:10.1038/srep31651)
Supplement: Supplementary Information [file srep31651-s1.doc]

**Up-regulation of miR-328-3p sensitizes non-small cell lung cancer to radiotherapy**

Wei Ma^*^, Chao-nan Ma^*^, nan-nan Zhou, Xian-dong Li, Yi-jie Zhang^#^

**Supplementary Table S1.** miRNA differentially expressed in recurrent NSCLC compared to corresponding non-cancerous tissue

Note: Positive and negative fold change scores means significant down regulation and up regulation, respectively, in tumors.

| No. | miRNA name | Mean fold change | P-value |
| --- | --- | --- | --- |
| 1 | miR-95 | 3.89 | 0.0151 |
| 2 | miR-66 | 2.96 | 0.0069 |
| 3 | miR-335 | 2.68 | 0.0051 |
| 4 | miR-181-3p | 2.41 | 0.0068 |
| 5 | miR-324 | 1.89 | 0.0394 |
| 6 | miR-126 | 1.75 | 0.0178 |
| 7 | miR-24 | 1.67 | 0.0076 |
| 8 | miR-787 | 1.52 | 0.0020 |
| 9 | miR-328-3p | -2.62 | 0.0096 |
| 10 | miR-155 | -2.51 | 0.0411 |
| 11 | miR-7 | -2.29 | 0.0260 |
| 12 | miR-483-3p | -2.17 | 0.0145 |
| 13 | Let-7g | -1.95 | 0.0279 |
| 14 | miR-505 | -1.88 | 0.0411 |
| 15 | miR-200c | -1.54 | 0.0157 |

**Supplementary table S2.** Potential target genes of miR-328 involved in cell function

| No.# | Gene name | Function |
| --- | --- | --- |
| 1 | CELF2 | pre-mRNA regulation |
| 2 | CTNND2 | E-cadherin suppressor |
| 3 | TRAPPC11 | Intracellular vesicle trafficking |
| 4 | ZNF131 | DNA binding transcription factor |
| 5 | EID1 | Transcription corepressor |
| 6 | PURG | DNA replication and transcription |
| 7 | KIF4B | Microtubule motor activity |
| 8 | HDGF | Cellular proliferation and differentiation |
| 9 | SNX1 | TGF-beta Receptor regulation |
| 10 | TTC3 | Ubiquitin-protein transferase activity |
| 11 | RAI14 | Cell-cell adhesion |
| 12 | MMP8 | Extracellular matrix |
| 13 | CUL4B | Cell cycle regulation and chromatin remodeling |
| 14 | SGPP1 | Multiple biolocial process |
| 15 | CCND1 | Cell cycle regulation |
